# Canine non-B, non-T NK lymphocytes have a potential antibody-dependent cellular cytotoxicity function against antibody-coated tumor cells

**DOI:** 10.1186/s12917-019-2068-5

**Published:** 2019-10-14

**Authors:** Yoseop Kim, Soo-Hyeon Lee, Cheol-Jung Kim, Je-Jung Lee, Dohyeon Yu, Soomin Ahn, Dong-Jun Shin, Sang-Ki Kim

**Affiliations:** 1 Department of Companion and Laboratory Animal Science, College of Industrial Science, Kongju National University, Yesan-gun, Chungnam, 32439 Republic of Korea; 2 Present Address: Research Institute, Vaxcell-Bio Therapeutics, Hwasun, Jellanamdo Republic of Korea; 3 Department of Integrated Life Science and Technology, Kongju National University, Yesan-gun, Chungnam, Republic of Korea; 4 Present Address: CHABiolab Co.,Ltd, Seongnam-si, Gyeonggi-do, Republic of Korea; 5 Department of Hemotology-Oncology, Chonnam National University Hwasun Hospital, Hwasun, Jeollanamdo Republic of Korea; 6 Institute of Animal Medicine, College of Veterinary Medicine, Gyeongsang National University, Jinju, Republic of Korea; 7 Research Institute for Natural Products, Kongju National University, Yesan-gun, Chungnam, 32439 Republic of Korea

**Keywords:** Natural killer cells, Canine, Antibody-dependent cellular cytotoxicity, Trastuzumab, Cetuximab

## Abstract

**Background:**

The antibody-dependent cellular cytotoxicity (ADCC) is a cell-mediated immune defense mechanism in which effector immune cells actively lyse antibody-coated target cells. The ADCC of tumor cells is employed in the treatment of various cancers overexpressing unique antigens, and only natural killer (NK) cells are known to be major effectors of antibody mediated ADCC activity. Canine NK cells are still defined as non-B, non-T large granular lymphocytes because of the lack of information regarding the NK cell-restricted specific marker in dogs, and it has never been demonstrated that canine NK cells have ADCC ability against tumor cells. In the present study, we investigated whether canine non-B, non-T NK cells have ADCC ability against target antibody-coated tumor cells, using cetuximab and trastuzumab, the only human antibodies reported binding to canine cancer cells.

**Results:**

Activated canine non-B, non-T NK cells (CD3^−^CD21^−^CD5^−^TCRαβ^−^TCRγδ^−^) for 13~17 days ex vivo showed ADCC ability against trastuzumab- or cetuximab-coated target tumor cells expressing various levels of human epidermal growth factor receptor 2 (HER-2) and epidermal growth factor receptor (EGFR). Trastuzumab and cetuximab induced significant ADCC responses of canine NK cells even in CMT-U334 and CF41.Mg cells expressing low levels of HER-2 and/or EGFR, as well as in SKBR3 and DU145 cells overexpressing HER-2 and/or EGFR. The trastuzumab-mediated ADCC activity of NK cells was significantly enhanced by treatment with rcIL-21.

**Conclusions:**

The results of this study suggest that canine non-B, non-T NK lymphocytes have a potential ADCC function and that combinational strategies of monoclonal antibodies with either cytokines, which activate NK cells in vivo, or adoptive transfer of NK cells may be a feasible method for amplifying the efficacy of immunotherapy against malignant cancers even with very low expression of target molecules in dogs.

**Electronic supplementary material:**

The online version of this article (10.1186/s12917-019-2068-5) contains supplementary material, which is available to authorized users.

## Background

The range of cancers observed in dogs is known to be as diverse as that observed in humans, and cancers in dogs and humans share many features, including histological and genetic molecular alterations, and biological behavior. Many conventional therapies applied to canine patients are nearly identical to those used to treat human patients [1]. Advances in conventional therapies, such as surgery, chemotherapy, and radiotherapy have contributed to the achievement of local control of the primary tumor, however, in general have failed to improve survival for cancer patients. Immunotherapy has emerged as an important addition to conventional cancer therapies [2]. In particular, natural killer (NK) cell-based immunotherapy and passive immunotherapy with targeted monoclonal antibodies have been the most successful therapeutic strategies for cancers in human [3–5].

NK cells are key components of the innate immune system, and mediate innate defenses against cancers and viral infections. NK cells are a powerful tool in cancer immunotherapy due to their robust effector functions. One of the potent effector mechanisms of NK cells is antibody-dependent cellular cytotoxicity (ADCC) mediated by antibody-coated target cells [4, 5]. The ADCC activity of NK cells is thought to play a crucial role in antitumor effects of therapeutic monoclonal antibodies for cancer [6, 7]. Only NK cells are known to be major effectors of antibody-mediated ADCC activity [6, 8]. The antitumor efficacy of target monoclonal antibody therapies has been shown to be NK cell-dependent [4, 5]. Clinical studies of NK cell-based immunotherapy combined with target monoclonal antibody therapies have shown significantly improved disease outcome in human cancer patients [9, 10]. However, the development of this approach in dogs has been precluded due to lack of information on canine NK cells and the lack of comparable therapeutic antibodies [11–13]. Canine NK cells can be defined as non-B, non-T large granular lymphocytes because of the lack of information regarding specific NK cell markers. Furthermore, it has never been demonstrated that canine NK cells have ADCC ability against antibody-coated tumor cells.

Therapeutically efficacious canine monoclonal antibodies are still not available, although several canine specific monoclonal antibodies are recently in various stages of development for canine cancers [14–17]. A recent study revealed significant homology between human and canine epidermal growth factor receptor (EGFR) and human epidermal growth factor receptor 2 (HER-2) and indicated that both molecules contain highly conserved epitopes for the therapeutic antibodies cetuximab and trastuzumab, with targeting inducing the tumoristatic effects of canine tumor cells overexpressing EGFR or HER-2 [18]. In the present study, we investigated whether the ADCC of canine NK cells was induced by antibody-coated cancer cells using cetuximab and trastuzumab, the only human antibodies reported to bind to canine cancer cells. The results of this study demonstrate that activated canine non-B, non-T NK lymphocytes have a potential ADCC function against antibody-coated tumor cells even with very low expression of target antigens.

## Results

### Expression of HER-2 and EGFR on the surface of target tumor cells

**Fig. 1:**
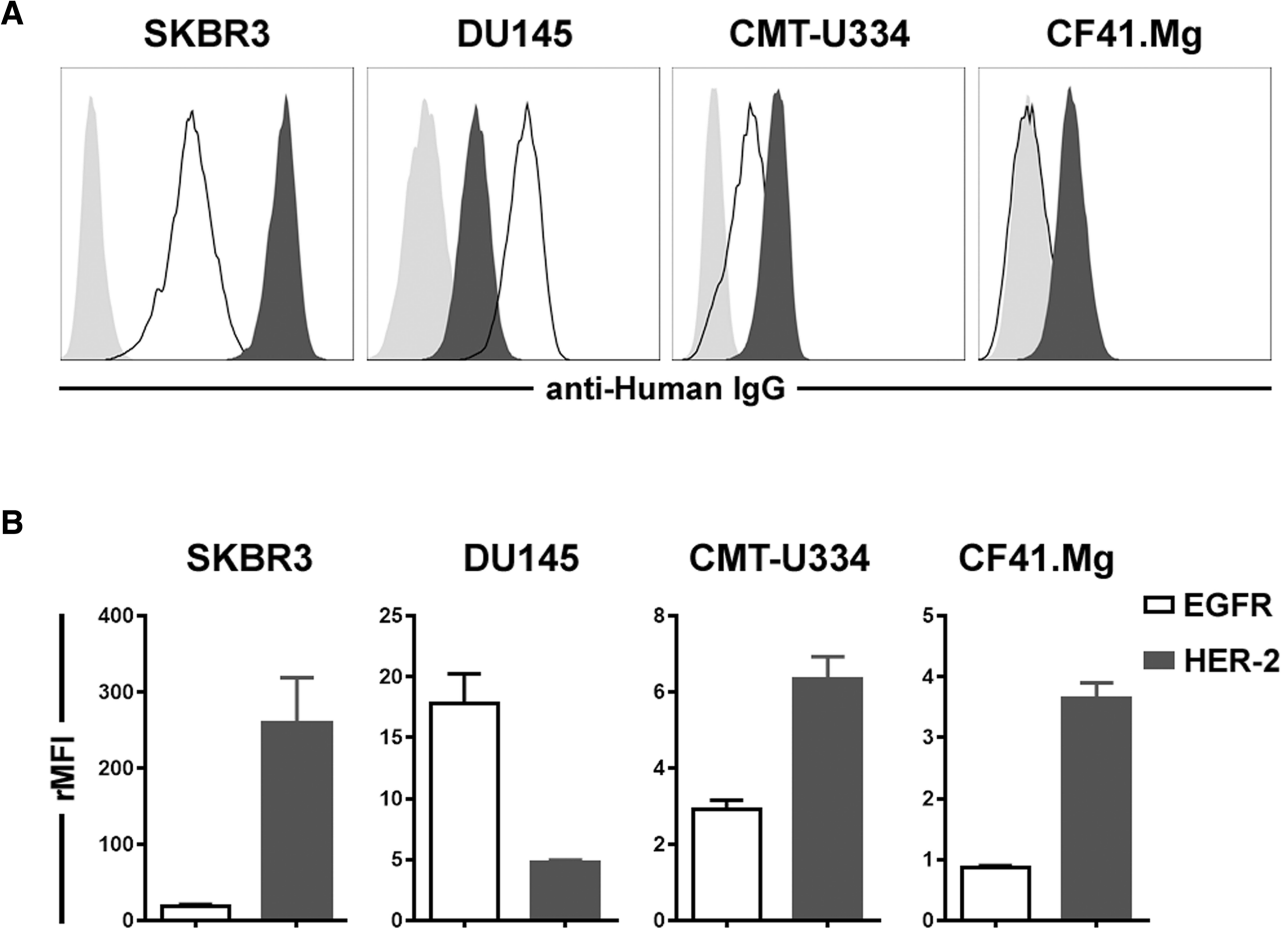
Expression levels of EGFR (black line) and HER-2 (black-filled histogram) on the surface of tumor cells measured by flow cytometry. **a** Representative flow cytometry data. The gray-filled histogram represents the isotype control. The data are representative of four independent experiments, each tested in triplicate. **b** Analysis of EGFR and HER-2 expression. Expression levels of EGFR and HER-2 represent the relative mean fluorescence intensity (rMFI). The results are shown as means ± standard deviation (SD) measured in triplicate from four independent experiments

To select the appropriate cells for the analysis of ADCC function in canine NK cells and confirm the antitumor effects of trastuzumab and cetuximab on canine tumor cells, the surface expression of HER-2 and/or EGFR was first investigated in canine tumor cell lines by flow cytometry. Four canine urinary bladder cancer cell lines, four mammary gland cancer cell lines, and canine thyroid adenocarcinoma (CTAC) cells were screened for their EGFR and HER-2 expression levels (Additional file 1: Methods). Based on the results of flow cytometry analysis, the CMT-334 and CF41.Mg cell lines were selected as the mammary gland tumor cells with the highest expression of HER-2 and/or EGFR among the nine cell lines tested for this study (Fig. 1 and Additional file 1: and Figure S1). SKBR3 and DU145 cells, which have been reported to highly express HER-2 and/or EGFR [19, 20], were used as positive controls. As shown in Fig. 1, the SKBR3 cells expressed high levels of HER-2 (relative mean fluorescence intensity, rMFI, 259.0 ± 120.4) and moderate levels of EGFR (rMFI = 18.4 ± 6.5). The DU145 cells expressed low levels of HER-2 (4.8 ± 0.4) and moderate levels of EGFR (17.8 ± 6.5). The CMT-U334 cells expressed low levels of HER-2 (6.3 ± 1.2) and low levels of EGFR (2.9 ± 0.5). CF41.Mg cells expressed low levels of HER-2 (3.7 ± 0.5) and did not express EGFR (0.9 ± 0.1) on their surface.

### Effects of trastuzumab and cetuximab on the viability, proliferation, and apoptosis of target tumor cells

**Fig. 2:**
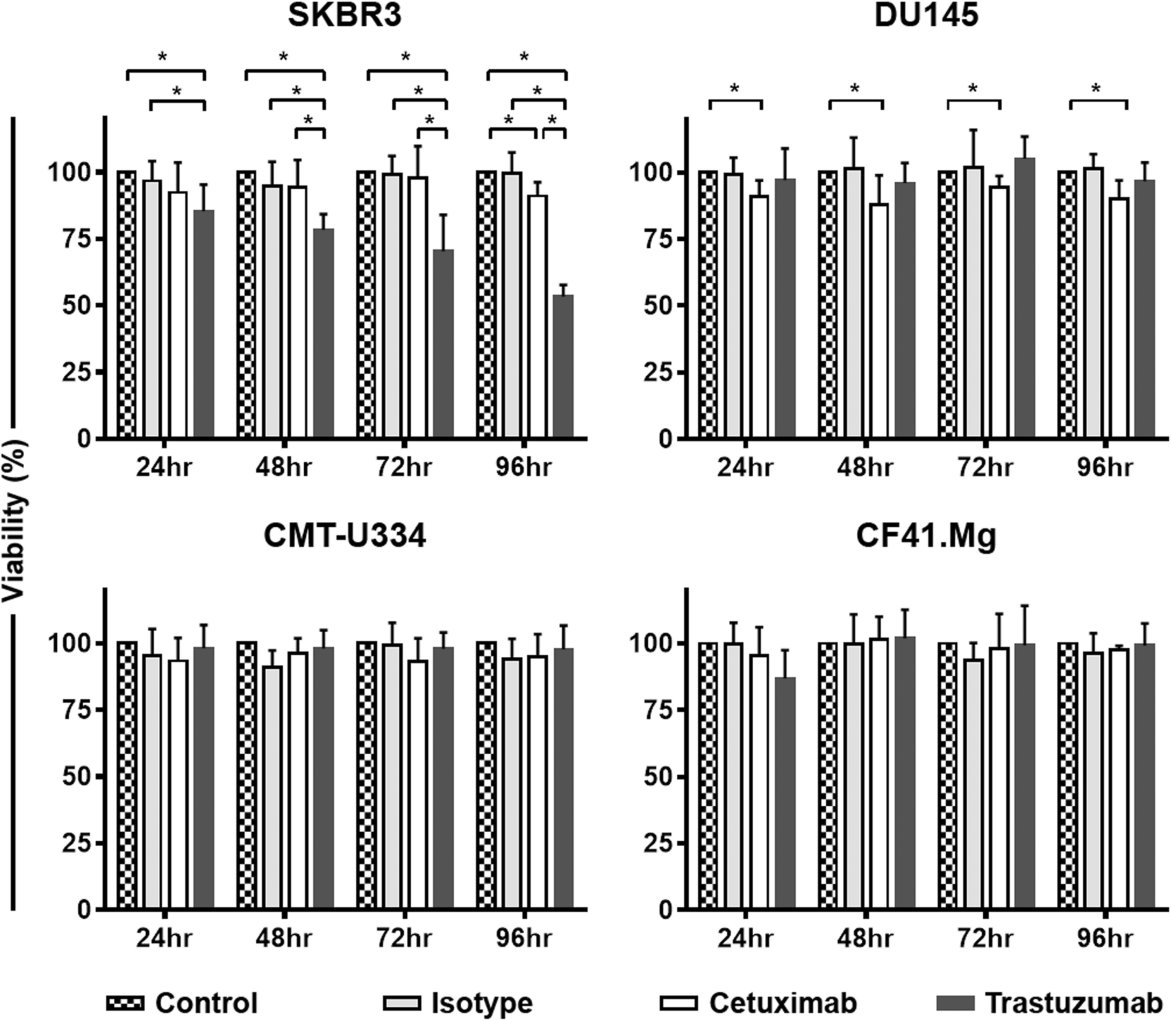
Effect of trastuzumab and cetuximab on the cell viability of tumor cells. The susceptibility of canine mammary gland tumor cells expressing EGFR and/or HER-2 to cetuximab and trastuzumab was investigated in a tetrazolium-based cell viability assay. SKBR3 and DU145 cells that had been reported to overexpress HER-2 and EGFR were used as positive controls. The viability of the tumor cells after treatment with trastuzumab, cetuximab, or human IgG isotype antibody was evaluated every 24 h for 96 h. Tumor cells incubated in media alone served as a control. The values represent the means ± standard deviation (SD) of five independent experiments, each tested in triplicate. *p < 0.05

To assess the direct anti-cancer effects of trastuzumab and cetuximab on canine tumor cells expressing low levels of EGFR and/or HER-2, the cell viability of CMT-U334 and CF41.Mg cells was analyzed after treatment with trastuzumab (10 μg/ml) or cetuximab (10 μg/ml). SKBR3 and DU145 cells, which have been reported to be sensitive to trastuzumab and/or cetuximab, were used as controls. The cell viability was not changed in CMT-U334 and CF41.Mg cells for 96 h after treatment with trastuzumab or cetuximab, whereas the cell viability of SKBR3 was significantly reduced in a time-dependent manner for 96 h after trastuzumab treatment compared with cells treated with human IgG isotype control antibody (99.5 ± 8.0%) or medium only (100.0 ± 0.0%) (*p* < 0.05). After 96 h of incubation with trastuzumab, the viability of SKBR3 cells was reduced to 53.4 ± 4.4%. Cetuximab did not inhibit the viability of SKBR3 cells until 72 h after treatment. However, the viability of SKBR3 cells was significantly decreased (91.1 ± 5.2%) after 96 h of cetuximab treatment compared with that of the IgG isotype control group (99.5 ± 8.0%) and media-only group (100.0 ± 0.0%) (*p* < 0.05). The viability of DU145 cells after treatment with cetuximab (90.1 ± 7.0%) was slightly reduced compared with that of the cells treated with human IgG isotype control antibody (101.3 ± 5.5) or media only (100.0 ± 0.0) (*p* < 0.05); however, no changes were observed in the viability of the cells after trastuzumab treatment (Fig. 2).

**Fig. 3:**
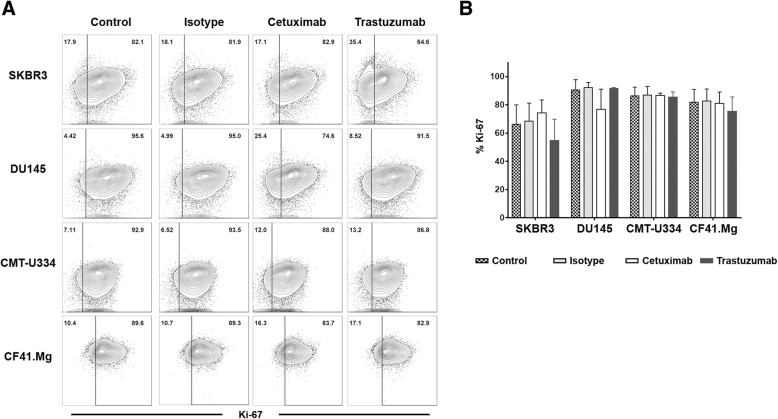
Effect of cetuximab and trastuzumab on the anti-proliferation of tumor cells expressing EGFR and/or HER-2. Expression of intracellular Ki-67, an indicator of cell proliferation, was analyzed within the target cells by flow cytometry 96 h after culture with cetuximab or trastuzumab treatment to investigate its inhibitory effect on the proliferation of canine tumor cells expressing EGFR and/or HER-2. The control groups were treated with media alone or human IgG isotype control antibody. **a** Representative flow cytometry data of intracellular Ki-67 expression. The data are representative of four independent experiments, each tested in triplicate. **b** The frequency of Ki-67-expressing cells within target tumor cells 96 h after culture with trastuzumab and cetuximab treatment. The values represent the means ± standard deviation (SD). The data are representative of four independent experiments, each tested in triplicate

**Fig. 4:**
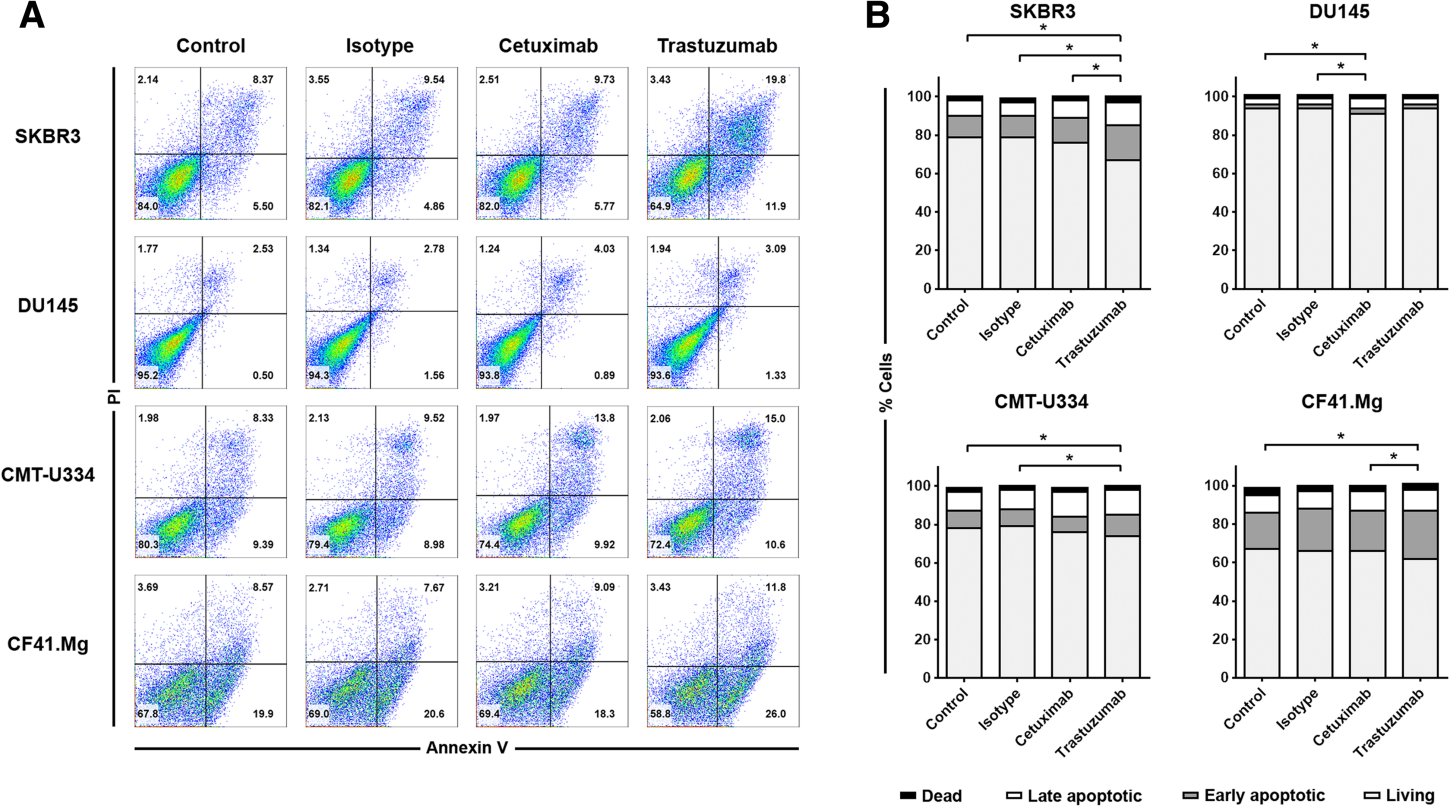
Effect of cetuximab and trastuzumab on the cell death of tumor cells expressing EGFR and/or HER-2. Target tumor cells were stained with annexin V/propidium iodide (PI) to analyze apoptosis of cells after culture of the cells in complete medium containing 10 μg/ml of cetuximab or 10 μg/ml of trastuzumab for 96 h. Control groups were treated with media alone or 10 μg/ml of human IgG isotype control antibody. **a** Representative flow cytometry data of annexin V/PI staining of cells from four independent experiments, each tested in triplicate. **b** The frequency of cell death 96 h after culture. The population of cells that was negative for both annexin V and PI was defined as living cells. Annexin V^+^/PI^*−*^ cells were classified as early apoptotic cells, and double-positive cells were classified as late apoptotic cells. Annexin V^*−*^/PI^+^ cells were classified as dead cells. The data are representative of four independent experiments, each tested in triplicate. *p < 0.05

To further investigate the growth-inhibitory effects of cetuximab and trastuzumab on canine cancer cells, the effects of both monoclonal antibodies on the proliferation and apoptosis of the target tumor cells were also examined. The expression of intracellular Ki-67 was analyzed to evaluate the cell proliferation by flow cytometry. Figure 3 shows the effects of cetuximab and trastuzumab on target cell proliferation. Neither antibody affected the proliferation of CMT-U334 and CF.41 Mg cells after 96 h of treatment. Although the difference was not statistically significant, trastuzumab tended to decrease the proliferation of SKBR3 (54.9 ± 15.0%) compared with that of the IgG isotype control group (68.4 ± 12.9%) and media-only group (66.3 ± 13.7%), and cetuximab tended to decrease the proliferation of DU145 (76.8 ± 14.3%) compared with that of the IgG isotype control group (92.1 ± 3.8%) and media-only group (90.6 ± 7.2%) after 96 h of treatment. The percentage of cells undergoing death was determined by the expression of propidium iodide (PI) and annexin V in all cells. Figure 4 shows the effects of cetuximab and trastuzumab on target cell apoptosis. After 96 h of treatment, trastuzumab enhanced apoptosis significantly in SKBR3 cells (33.2 ± 11.3%) compared with that in the cetuximab group (24.2 ± 8.2%), the isotype IgG antibody group (21.0 ± 8.4%), and the media-only group (21.0 ± 7.1%) (*p* < 0.05). Trastuzumab increased apoptosis slightly in CMT-U334 cells (26.2 ± 9.5%) compared with that in the isotype IgG antibody group (20.9 ± 7.9%) and media-only group (22.2 ± 8.2%) (*p* < 0.05). In CF41.Mg cells, there was no significant difference compared with the isotype IgG antibody (34.0 ± 8.3%), but trastuzumab significantly increased apoptosis (38.4 ± 6.8%) compared with the cetuximab group (33.9 ± 7.1%) and media-only group (32.5 ± 8.4%) (*p* < 0.05). Cetuximab significantly increased apoptosis only in DU145 cells (9.1 ± 3.2%) compared to the isotype IgG antibody group (6.4 ± 1.4%) and media-only group (6.2 ± 2.1%) (*p* < 0.05).

### ADCC activity of canine non-B, non-T NK cells in response to trastuzumab- or cetuximab-coated tumor cells

**Fig. 5:**
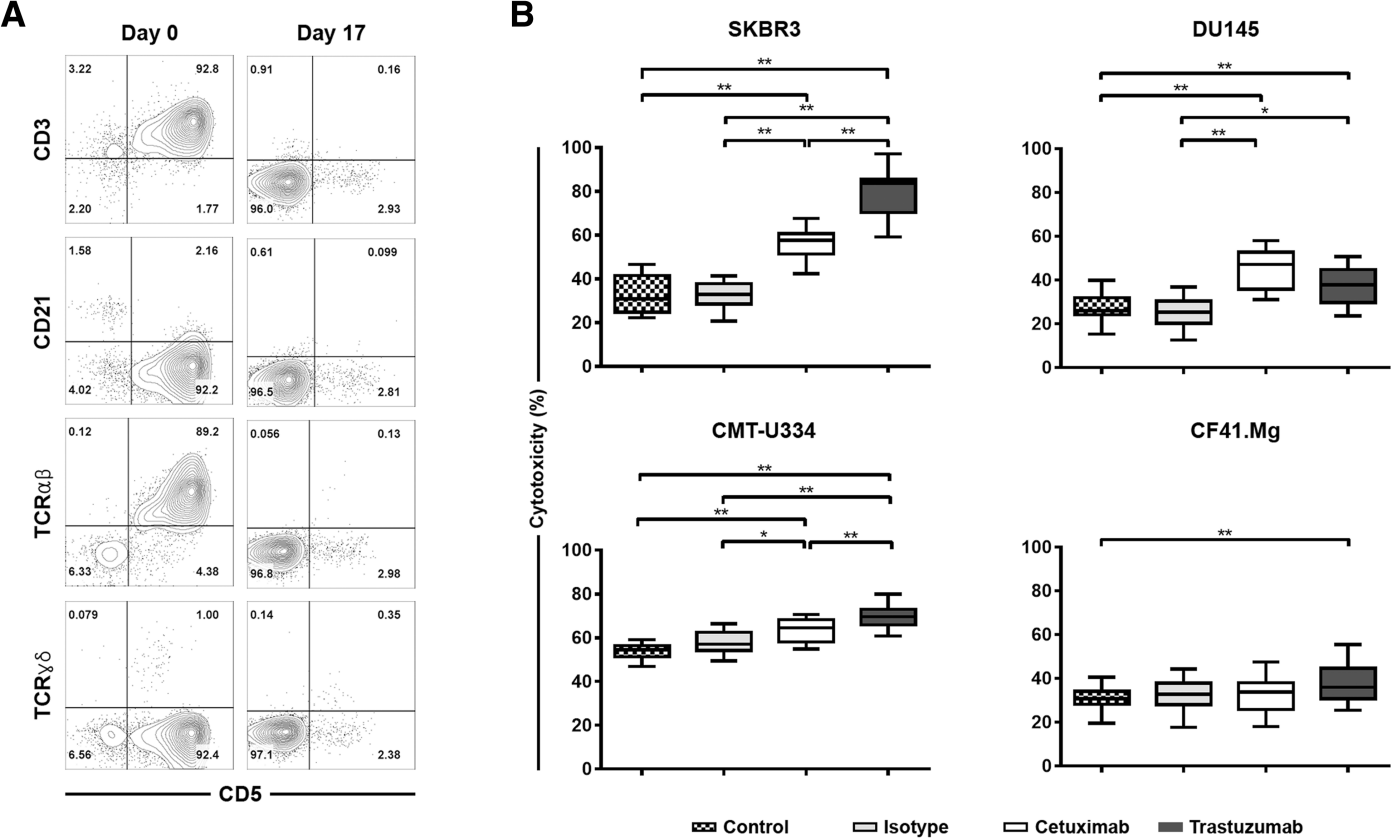
Phenotype and antibody-dependent cellular cytotoxicity (ADCC) of cultured canine NK cells. **a** Phenotypic characteristics of expanded non-B, non-T NK lymphocytes. PBMCs (day 0) and expanded NK cells (day 17) were stained with anti-CD3, −CD5, −CD21, −TCRαβ, and -TCRγδ and were analyzed via flow cytometry. Expanded NK cells (CD3^−^CD21^−^CD5^−^TCRαβ^−^TCRγδ^−^) exhibiting over 90% purity after culture were used to evaluate the ADCC effects of such cells in canine tumor cells. **b** The ADCC ability of expanded NK cells against target tumor cells pretreated with trastuzumab or cetuximab. The 4-h cytotoxicities of NK cells were measured at a 4:1 effector-to-target (E:T) ratio from triplicate reactions and 12 different donors. The cytotoxicity of NK cells against tumor cells pretreated with media alone or human isotype IgG antibody served as controls. The median, first (Q1) and third (Q3) quartiles, and the minimum and maximum are shown. *p < 0.05, **p < 0.01

Canine non-B, non-T NK cells were selectively expanded for 13~17 days ex vivo until the purity of the cells reached greater than 90% depending on the donor. The phenotype of most of these expanded cells was CD3^−^CD21^−^CD5^−^TCRαβ^−^TCRγδ^−^, and the purity of the cells was 93.4 ± 2.7% (Fig. 5a). The mRNA expression levels of NK-related molecules, including CD16, in the expanded cells were the same as those in our previous reports (data not shown) [13, 21, 22]. To assess the ADCC ability of canine non-B, non-T NK cells against the antibody-coated target tumor cells, the 4-h cytotoxicity of NK cells was investigated after co-culture with trastuzumab- or cetuximab-coated CMT-U334, CF41.Mg, SKBR3, and DU145 cells at a 4:1 E:T ratio. As shown in Fig. 5b, trastuzumab significantly enhanced the cytotoxic activities of canine NK cells against not only SKBR3 expressing high levels of HER-2 but also DU145 and CMT-U334 expressing low levels of HER-2 (*p* < 0.01). Canine NK cells also showed significantly higher cytotoxicity in trastuzumab-coated CF41.Mg cells expressing very low levels of HER-2 than in control cells not coated with antibodies (*p* < 0.01). The isotype IgG control antibody did not influence the cytotoxicity of NK cells against all four target tumor cells compared with control cells not treated with antibody. With the exception of CF41.Mg cells that do not express EGFR, cetuximab also significantly increased the cytotoxicity of NK cells in DU145, SKBR3, and CMT-U334 cells expressing various levels of EGFR (*p* < 0.01).

### Augmentation of ADCC activity and interferon (IFN)-γ production in NK cells in response to trastuzumab-coated SKBR3 cells by IL-21

**Fig. 6:**
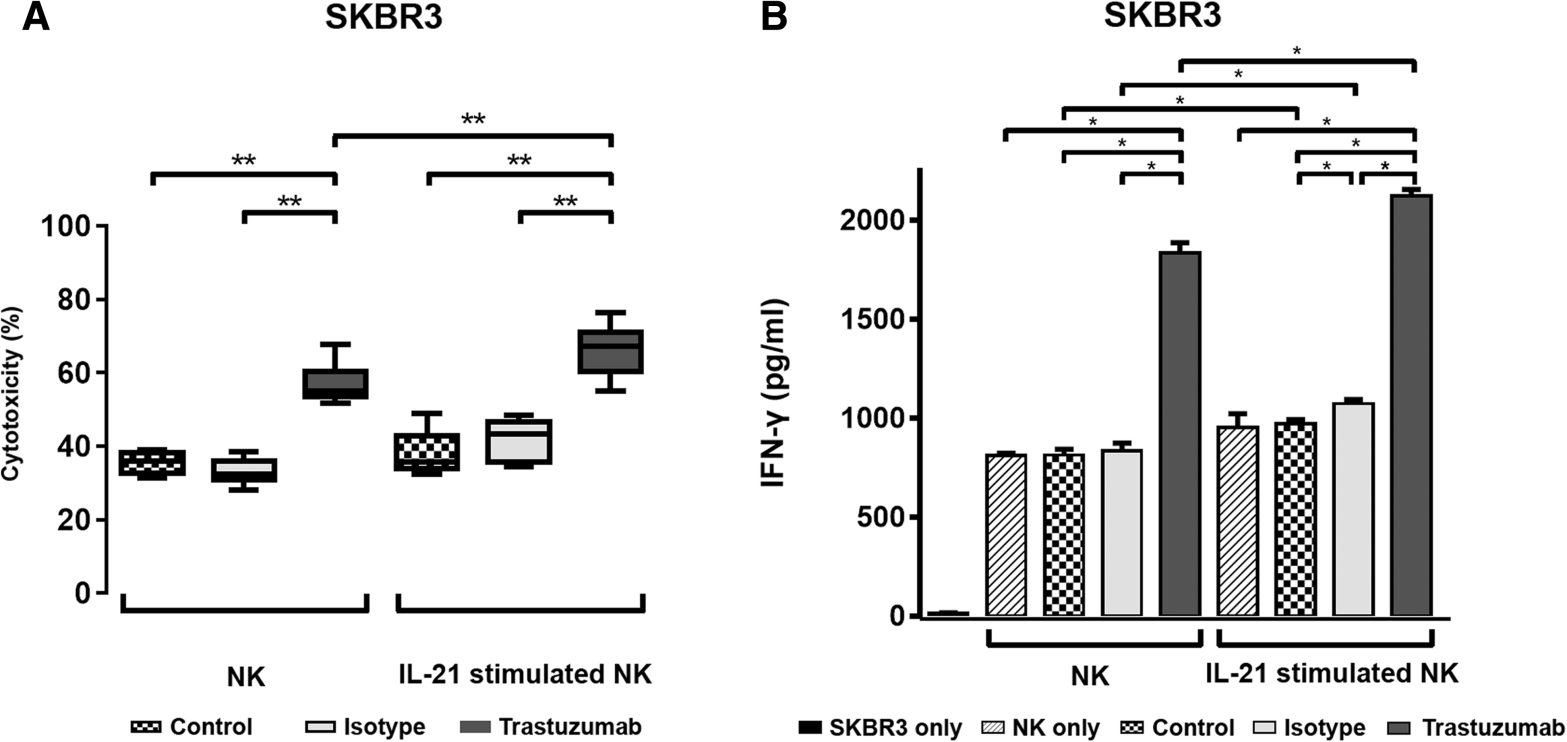
Effects of IL-21 stimulation on ADCC activity and interferon (IFN)-γ production of cultured canine NK cells. To determine the capacity of additional IL-21 stimulation on the enhancement of effector function, cultured NK cells were additionally treated with rcIL-21 for 2 days prior to harvest (additional IL-21), and the cytotoxicity and IFN-γ production against trastuzumab-coated SKBR3 cells were compared with cultured NK cells without additional IL-21 stimulation (NK). **a** The 4-h cytotoxicities of NK cells against SKBR3 cells pretreated with trastuzumab were measured at a 4:1 E:T ratio. The cytotoxicity of NK cells against SKBR3 cells pretreated with media alone (Control) or the human isotype IgG antibody (Isotype) served as the controls. The median, first (Q1) and third (Q3) quartiles, and the minimum and maximum are shown from triplicate reactions and five different donors. **b** Production of IFN-γ in cultured NK cells in response to SKBR3 pretreated with trastuzumab. IFN-γ production was assessed in the culture supernatants by ELISA. Control conditions consisted of the culture supernatant from SKBR3 cells (SKBR3 only), NK cells cultured alone (NK only), or NK cells cultured with SKBR3 cells with (Isotype) or without (Control) pretreatment of human IgG isotype antibody. The results are shown as means ± standard deviation (SD) from triplicate reactions and four different donors. *p < 0.05, **p < 0.01

To determine the effects of additional IL-21 stimulation on the effector functions of NK cells against trastuzumab-coated tumor cells, the cultured NK cells were further treated with IL-21 for 2 days before harvesting on day 14. SKBR3 cells expressing high levels of HER-2 were treated with trastuzumab, isotype IgG antibody, and media only and then were co-cultured with NK cells that had been treated with or without IL-21. As shown in Fig. 6a, 4-h cytotoxicity analysis revealed that the trastuzumab-mediated ADCC activity of NK cells against SKBR3 cells was enhanced significantly by cultured NK cells pretreated with IL-21 (66.1 ± 6.9%) compared with those without additional IL-21 stimulation (57.1 ± 5.2%) (*p* < 0.05). Compared with control cells not treated with antibody, the isotype IgG control antibody did not affect the cytotoxicity of NK cells regardless of IL-21 stimulation against SKBR3 cells. We next assessed the IFN-γ production of cultured NK cells stimulated with or without additional IL-21 in response to trastuzumab- or isotype IgG antibody-coated and uncoated SKBR3 cells after a 24-h co-culture at a 10:1 E:T ratio. As shown in Fig. 6b, the production of IFN-γ in NK cells was, with or without additional IL-21 stimulation, significantly increased in response to trastuzumab-coated SKBR3 cells compared with isotype IgG antibody-coated or antibody-untreated SKBR3 cells (*p* < 0.05). Additional stimulation of cultured NK cells with IL-21 further increased the production of IFN-γ in response to trastuzumab-coated SKBR3 cells (1832.7 ± 92.9 pg/ml) compared to NK cells untreated with IL-21 (2121.3 ± 58.0 pg/ml) (*p* < 0.05).

## Discussion

In this study, we investigated whether canine non-B, non-T NK cells possess ADCC function against antibody-coated tumor cells, using cetuximab and trastuzumab, the human antibodies reported to bind to canine cancer cells expressing EGFR and HER-2. EGFR (erbB1) and HER-2 (erbB2) are members of the ErbB receptor tyrosine kinase family that consists of four members, and the dimerization of the receptor that occurs after ligand binding to the extracellular domain finally controls various biological responses, such as the proliferation, survival, and migration of cells [23]. The overexpression of EGFR and HER-2 in different types of canine malignancies, such as mammary gland tumors [24, 25], osteosarcoma [26], gastric tumors [27], and brain tumors [28], has been reported. The molecular structures and biological functions of canine EGFR and HER-2 were found to be very similar to those of human EGFR and HER-2 [18]. Cetuximab, a chimeric IgG1 antibody against EGFR, and trastuzumab, a humanized IgG1 antibody against HER-2, are popular therapeutic antibodies that significantly improved the clinical outcome in EGFR- and HER-2-overexpressing human cancers, respectively [29]. A recent report demonstrated the direct tumoristatic effects of cetuximab and trastuzumab on canine mammary carcinoma cells expressing EGFR and HER-2 [18]. In the present study, we confirmed that cetuximab and trastuzumab antibodies were bound to canine EGFR and HER-2, respectively, on mammary gland carcinoma cells (Fig. 1). Although trastuzumab induced very marginal levels of apoptosis in both cell lines (Fig. 4), cetuximab and trastuzumab did not show effects on the cell growth and proliferation of CF41.Mg and CMT-U334 cells, both of which express low levels of HER-2 and EGFR (Figs. 2 and 3), These results are in agreement with human studies showing that the direct anti-cancer effects of cetuximab and trastuzumab were not observed in tumor cells expressing low levels of EGFR and HER-2, respectively [19, 30].

The anti-tumor efficacy of tumor targeting monoclonal antibodies are shown to be NK cell-dependent, because the NK cell-mediated ADCC activities of monoclonal antibodies are crucial for their anti-cancer effects [5–8]. Canine NK cells are still defined as non-B, non-T large granular lymphocytes because of the lack of information regarding the NK cell-restricted specific marker in dogs, although CD3^−^CD21^−^ lymphocytes that express NKp46 (NCR1) are thought be a population of canine NK cells [31, 32]. In our previous studies, we selectively expanded canine non-B, non-T NK lymphocytes (CD3^−^CD21^−^CD5^−^CD4^−^TCRαβ^−^TCRγδ^−^) ex vivo from the PBMCs of healthy dogs, and characterized them phenotypically and functionally [13]. These expanded NK lymphocytes expressed NK cell-related genes, including CD16 (FcγRIII) which is important for the ADCC function of NK cells [13, 21, 22, 33, 34]. In the present study, canine NK cells were expanded for 13~17 days under the same culture conditions used in our previous study (Fig. 5a) [13]. Most of canine non-B, non-T NK cells cultured in this condition expressed NKp46 on their surface (Additional file 1: Figure S2). We investigated the ADCC function of cultured non-B, non-T NK cells against the target tumor cells with various expression levels of HER-2 and/or EGFR. Trastuzumab and cetuximab induced significant ADCC responses of canine NK cells in tumor cells expressing HER-2 and/or EGFR, regardless of the degree of expression (Fig. 5b), suggesting that FcγRIII expressed on the surface of cultured canine NK cells can bind to the Fc constant regions of the human antibodies as reported by Bergeron et al. [35]. The canine NK cells showed significant ADCC capability against trastuzumab- and cetuximab-coated SKBR3 and DU145 overexpressing HER-2 and/or EGFR (Fig. 5b). Although both SKBR3 and DU145 are human-derived cancer cells, the results of significantly enhanced cytotoxic effects of NK cells against antibody-coated cells compared to controls may be sufficient to demonstrate ADCC function of canine NK cells. Notably, trastuzumab and cetuximab could efficiently stimulate the ADCC activity of canine NK cells even against tumor cells, which weakly express HER-2 and/or EGFR. Trastuzumab induced significant ADCC responses in CMT-U334 and CF41.Mg cells expressing low levels of HER-2. However, the magnitude of the difference in NK cell cytotoxicity against trastuzumab-uncoated and -coated CF41.Mg cells was too small to make biological significance. Cetuximab significantly stimulated ADCC activities in EGFR low-expressing CMT-U334 cells but not in CF41.Mg cells that do not express EGFR (Fig. 5b). These results are consistent with the findings in human studies showing that trastuzumab induces ADCC in HER-2-non-amplified breast cancer cells [7, 36]. On the other hand, trastuzumab and cetuximab did not stimulate ADCC activity of cultured NK cells against canine NK-sensitive CTAC (canine thyroid adenocarcinoma) cells that do not express HER-2 and EGFR (Additional file 1: Figure S1 and S3). These results might indicate that a threshold level of HER-2 or EGFR expression is required for initiation of trastuzumab- or cetuximab-mediated ADCC enough to make a therapeutic difference [7].

Taken together, the results of this study demonstrate that activated canine non-B, non-T NK lymphocytes have a potential ADCC function, and trastuzumab and cetuximab, which have a human IgG1 backbone, can induce strong ADCC activity of these cells against HER-2- and EGFR-expressing tumor cells, respectively. Furthermore, canine NK lymphocytes are capable of ADCC function mediated by trastuzumab and cetuximab even in tumor cells with very low expression of HER-2 and EGFR. Recently, several canine specific anti-CD20 monoclonal antibodies and a chimeric version of anti-EGFR antibody are in various stages of development for treatment of canine cancers [14–17]. Further clinical research should focus on the combinational strategies of these canine antibodies with therapies to enhance NK cell function for canine cancer patients.

## Conclusion

The results of this study suggest that canine non-B, non-T NK lymphocytes have a potential ADCC function and that combinational strategies of monoclonal antibodies with either adoptive transfer of NK cells or cytokines like interleukin-15, which activate NK cells in vivo, may be a feasible method for amplifying the efficacy of immunotherapy against malignant cancers in dogs.

## Methods

### Cell lines and monoclonal antibodies

CMT-U334 cells (canine mammary gland tumor cells) were kindly provided by Dr. Eva Hellmen (Swedish University of Agricultural Sciences, Uppsala, Sweden). CF41.Mg (canine mammary gland tumor cells), SKBR3 (human breast carcinoma cells), DU145 (human prostate cancer cells), and K562 cells were obtained from the American Type Culture Collection (ATCC, Manassas, VA, USA). Cetuximab (Erbitux®), a chimeric IgG1 anti-ErbB-1 (EGFR) monoclonal antibody, was obtained from Merck KGaA (Darmstadt, Germany), and trastuzumab (Herceptin®), a humanized IgG1 monoclonal anti-ErbB-2 (HER-2) antibody, was from Roche (Hertfordshire, United Kingdom). The concentrations of cetuximab and trastuzumab used for all analyzes in this study were 10 μg/ml as determined by the binding assay (Additional file 1: Figure S4 and Methods).

### Animals and blood collection

Peripheral blood was obtained from nine healthy beagle dogs that were kept at the animal center of Kongju National University for research or educational purposes. All dogs had previously received routine vaccinations and had been dewormed regularly. Blood samples were collected from the jugular vein of each dog into 10 ml of sodium-heparin tubes (Becton Dickinson, Franklin Lakes, NJ, USA). The use of animals for this study was approved by the Institutional Animal Care and Use Committee of Kongju National University (KNU_2017–03). All dogs used in the study were adopted by students after the research was completed.

### Expansion of canine NK cells

Peripheral blood mononuclear cells (PBMCs) were isolated by discontinuous density gradient centrifugation, and canine NK cells were selectively expanded with a methods detailed in previous reports [13, 21]. In brief, isolated PBMCs (3.5 × 10^6^) were incubated with 125 Gy-irradiated K562 cells (0.5 × 10^6^) in complete RPMI-1640 medium (WELGENE, Seoul, Korea) supplemented with 10% FBS (WELGENE), 100 units/ml of penicillin, 100 μg/ml of streptomycin (WELGENE), 100 IU/ml of human interleukin (IL)-2 (PeproTec, Rocky Hill, NJ, USA), and 10 IU/ml of recombinant canine IL (rcIL)-15 (in house). Cells were stimulated with 5 ng/ml of rcIL-21 (R&D Systems, Minneapolis, MN, USA) for the first 7 days of culture. Fresh medium with rhIL-2 and rcIL-15 was provided every other day [13, 21]. To evaluate the capacity of IL-21 to enhance the ADCC activity of expanded NK cells, the cultured NK cells were additionally stimulated with rcIL-21 (R&D Systems) for 2 days before harvest on days 12~15, and the production of IFN-γ and the cytotoxicity against trastuzumab-coated SKBR3 cells were evaluated. The purity of the cultured NK cells was examined by flow cytometry as previously described [13, 21]. Cells were stained with FITC-conjugated anti-dog CD3 (clone CA17.2A12), APC-conjugated anti-dog CD5 (clone YKIX322.3), RPE-conjugated anti-dog CD21 (clone CA2.1D6) (all from Bio-Rad, Hercules, CA, USA), unconjugated TCRαβ (clone CA15.8G7), and TCRγδ (clone CA20.8H1) (both from Peter Moor, UC Davis, CA, USA). For TCRαβ and TCRγδ, Pacific Blue-conjugated goat anti-mouse IgG secondary antibody (Invitrogen, Carlsbad, CA, USA) was added.

### Analysis of EGFR/HER-2 expression in tumor cells

Expression of EGFR and HER-2 protein on tumor cells was assessed by flow cytometry. The tumor cells (2 × 10^5^) were incubated with 10 μg/ml of cetuximab (Merck KGaA) or trastuzumab (Roche) for 15 min on ice. The cells were then washed three times with flow cytometry (FACS) buffer (phosphate-buffered saline, 5% bovine serum albumin) and incubated with 2 μl of Alexa Fluor 488-conjugated goat anti-human IgG antibody (Southern Biotech, Birmingham, AL, USA) for 15 min on ice. After washing twice with FACS buffer, FACS analysis was performed using the FACSCalibur flow cytometer (Becton Dickinson). Data were analyzed with FlowJo software (Version 10.4.1.; FlowJo, LLC, Ashland, OR, USA).

### Cell viability assay

An EZ-Cytox Cell Viability Assay kit (ItsBio, Seoul, Korea) was used to determine the cytotoxic effects of cetuximab and trastuzumab against CMT-U334, SKBR3, DU145, and CF41.Mg cells for 96 h. Cells (4 × 10^4^/well) were cultured in a 96-well flat-bottom plate in triplicate overnight under standard culture conditions. After washing the cells twice with RPMI-1640 or DMEM medium supplemented with 10% FBS and antibiotics, we exposed the cells to 10 μg/ml of cetuximab or 10 μg/ml of trastuzumab for 24, 48, 72, and 96 h, followed by washing twice with medium. The control groups were treated with medium alone or 10 μg/ml of purified human IgG isotype control antibody (Novus Biologicals, Littleton, CO, USA). After adding 10 μl of WST-1 (ItsBio) to 150 μl of medium per well, the plates were incubated at 37 °C for 30 min and were placed on ice for 5 min to stop the reaction. The absorption at 450 nm was measured using the Infinite M200 PRO (Salzburg Umgebung, Salzburg, Austria) device to determine the amount of formazan produced by live cells.

### Cell proliferation and apoptosis assay

The effects of cetuximab and trastuzumab on the proliferation and apoptosis of tumor cells were determined by flow cytometry. CMT-U334 (2 × 10^5^), SKBR3 (4 × 10^5^), DU145 (4 × 10^5^), and CF41.Mg (4 × 10^5^) cells were seeded in 100-mm culture dishes and cultured in triplicate overnight under standard culture conditions. After washing the cells twice with medium, we incubated the cells in complete medium containing 10 μg/ml of cetuximab or 10 μg/ml of trastuzumab for 96 h. The control groups were treated with medium alone or 10 μg/ml of human IgG isotype control antibody (Novus Biologicals). After harvesting the cells and following cell permeabilization using a Foxp3/Transcription factor staining buffer set (eBioscience, San Diego, CA, USA), we measured the protein levels of Ki-67 molecules, an indicator of cell proliferation, by intracellular staining using a PE-Cyanine7-labeled mAb against Ki-67 (eBioscience). Isotype controls were run in parallel. The apoptosis of cells was analyzed using the FITC annexin V/dead cell apoptosis kit (Invitrogen) according to the manufacturer’s instructions. Flow cytometry analyses were performed using a FACSAria flow cytometer (Becton Dickinson). The data were analyzed using FlowJo software (FlowJo).

### NK cell cytotoxicity and ADCC assay

The EZ-Cytox Cell Viability Assay kit (ItsBio) was used to measure the 4-h cytotoxicity and ADCC activities of cultured NK cells, as previously described [21, 34]. Target tumor cells (4 × 10^4^/well) were seeded in a 96-well flat-bottom plate in triplicate and were cultured overnight under standard culture conditions. The next day, the target tumor cells were washed and exposed to media alone, 10 μg/ml purified human IgG isotype antibody (Novus Biologicals), 10 μg/ml cetuximab, or 10 μg/ml trastuzumab at 37 °C for 30 min. After washing the cells twice, we cultured the cells with expanded canine NK cells at a 4:1 effector-to-target (E:T) ratio at 37 °C for 3 h. After adding 10 μl of WST-1 (ItsBio) to the well, the plates were incubated at 37 °C for 1 h and placed on ice for 5 min to stop the reaction. The absorbance at 450 nm was measured using the Infinite M200 PRO (Salzburg Umgebung). The percent of cytotoxicity was calculated using the following equation: 100% − 100 × [*A*_450_ of effector cell-treated target cells − *A*_450_ of effector cells (background of effector cells)] / [*A*_450_ of target cells − *A*_450_ of target cells with no WST-1 (background of target cells)].

### Enzyme-linked immunosorbent assay (ELISA)

IFN-γ production in expanded NK cells in response to SKBR3 coated with trastuzumab was analyzed by ELISA as described previously [13, 21]. Target cells (2 × 10^4^) were seeded in a 96-well microplate in triplicate and cultured at 37 °C overnight. The next day, the cells were exposed to 10 μg/ml of human IgG isotype antibody or 10 μg/ml of trastuzumab at 37 °C for 30 min. The plate was then washed with medium, and expanded NK cells (2 × 10^5^) cells were co-cultured with target cells at a 10:1 E:T ratio without cytokines. After 24 h of co-culture, the cell-free culture supernatants were harvested and analyzed for IFN-γ production using DuoSet canine IFN-γ kits (R&D Systems, Minneapolis, MN, USA) according to the manufacturer’s instructions. The cell-free supernatant from SKBR3 and NK cells cultured alone for 24 h was used as the control.

### Statistical analysis

All statistical analyses were carried out using SPSS (Version 24.0; IBM Corp, Armonk, NY, USA). The significance of the differences between the groups was determined using the non-parametric Kruskal–Wallis test followed by post hoc comparison using the Dunn test. The Mann–Whitney *U* test was used for comparisons across two groups. A *p* value < 0.05 was deemed to indicate statistical significance.

## Additional file

Additional file 1: **Figure S1.** Expression levels of EGFR and HER-2 on the surface of canine tumor cells. **Figure S2.** Expression of NKp46 on cultured non-B, non-T (CD3^−^ CD5^−^ CD21^−^) NK lymphocytes. **Figure S3.** The ADCC ability of expanded canine NK cells against trastuzumab- or cetuximab-coated canine thyroid adenocarcinoma (CTAC) cells that do not express HER-2 and EGFR. **Figure S4.** Binding of trastuzumab and cetuximab to SKBR3 cells by flow cytometry. **Methods.** Cell lines and monoclonal antibody, and binding assay for trastuzumab and cetuximab. (DOCX 690 kb)

## Data Availability

The datasets used and/or analyzed during the current study available from the corresponding author on reasonable request.

## Abbreviations

ADCC: Antibody-dependent cellular cytotoxicity
CTAC: Canine thyroid adenocarcinoma
EGFR: Epidermal growth factor receptor
ELISA: Enzyme-linked immunosorbent assay
FACS: Flow cytometry
HER-2: Human epidermal growth factor receptor 2
NK cells: Natural killer cells
PBMCs: Peripheral blood mononuclear cells
PI: Propidium iodide
rcIL: Recombinant canine interleukin
