# Supplementary material for: Canine non-B, non-T NK lymphocytes have a potential antibody-dependent cellular cytotoxicity function against antibody-coated tumor cells
Source: BMC Vet Res. 2019 Oct 14;15:339. doi: 10.1186/s12917-019-2068-5 (PMC6790994; doi:10.1186/s12917-019-2068-5)
Supplement: Supplementary file 1 — Figure S1. Expression levels of EGFR and HER-2 on the surface of canine tumor cells. Figure S2. Expression of NKp46 on cultured non-B, non-T (CD3− CD5− CD21−) NK lymphocytes. Figure S3. The ADCC ability of expanded canine NK cells against trastuzumab- or cetuximab-coated canine thyroid adenocarcinoma (CTAC) cells that do not express HER-2 and EGFR. Figure S4. Binding of trastuzumab and cetuximab to SKBR3 cells by flow cytometry. Methods. Cell lines and monoclonal antibody, and binding assay for trastuzumab and cetuximab. (DOCX 690 kb) [file 12917_2019_2068_MOESM1_ESM.docx]

**Supplementary information**

**Canine non-B, non-T NK lymphocytes have a potential antibody-dependent cellular cytotoxicity function against antibody-coated tumor cells**

Yoseop Kim^1,a^, Soo-Hyeon Lee^2,a^, Cheol-Jung Kim^1^, Je-Jung Lee^3^, Dohyeon Yu^4^, Soomin Ahn^4^, Dong-Jun Shin^1,5,*^, Sang-Ki Kim^1,2,5,*^

^1^Department of Laboratory and Companion Animal Science, College of Industrial Science, Kongju National University, Yesan-gun, Chungnam, Republic of Korea

^2^Department of Integrated Life Science and Technology, Kongju National University, Yesan-gun, Chungnam, Republic of Korea

^3^Department of Hemotology-Oncology, Chonnam National Univresity Hwasun Hospital, Hwasun, Jeollanamdo, Republic of Korea

^4^Institute of Animal Medicine, College of Veterinary Medicine, Gyeongsang National University, Jinju, Republic of Korea

^5^Research Institute for Natural Products, Kongju National University, Yesan-gun, Chungnam, Republic of Korea

*** Corresponding author :** Correspondence and requests for materials should be addressed to S-K Kim (email: [sangki@kongju.ac.kr](mailto:sangki@kongju.ac.kr)) or D-J Shin (email: [shin-phd@daum.net](mailto:shin-phd@daum.net)).

^a^ These authors contributed equally to this study.

^[[1]](#footnote-1)^

**Supplementary Figures and Legends**


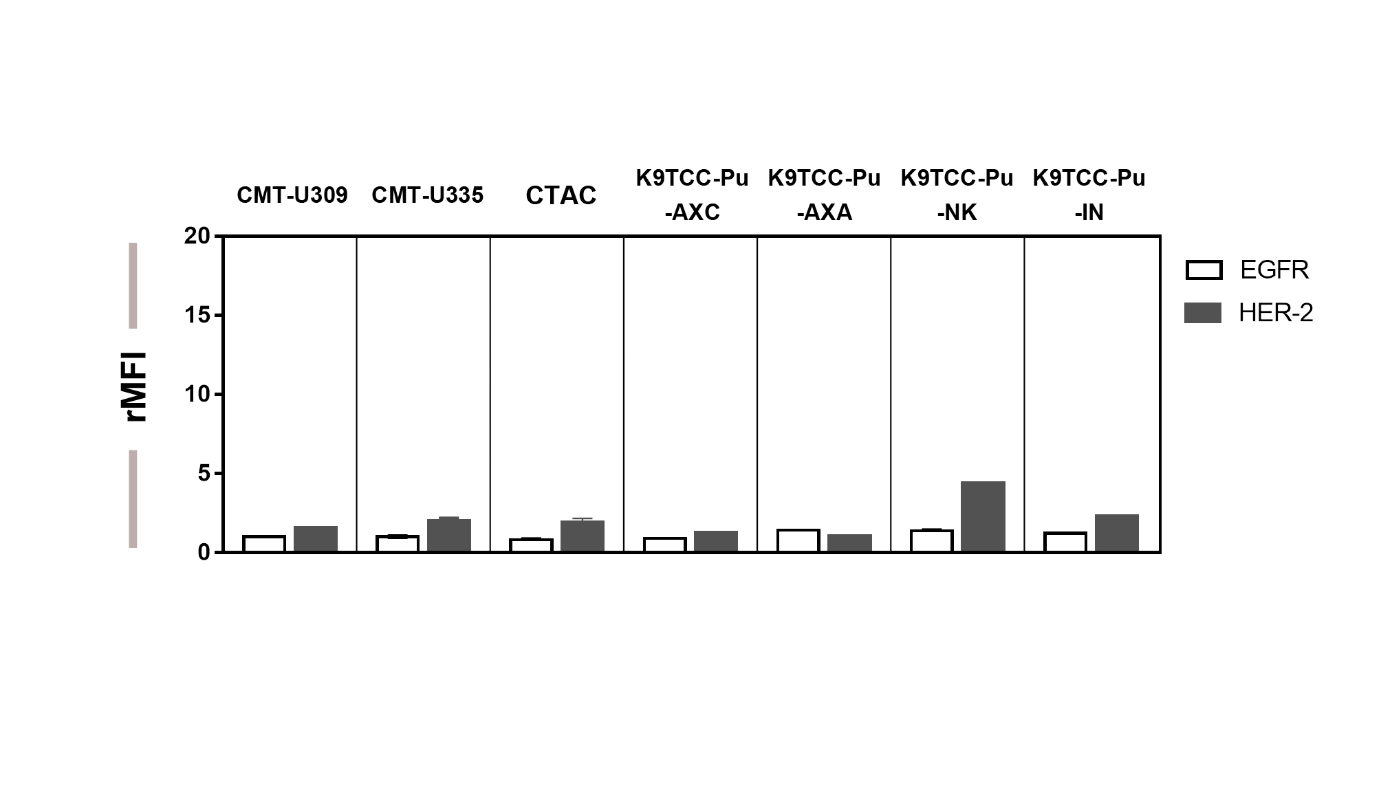


**Supplementary Fig. S1.** Expression levels of EGFR (black line) and HER-2 (black-filled histogram) on the surface of canine mammary gland tumor cells (CMT-U309, CMT-U336), canine thyroid adenocarcinoma (CTAC), and canine urinary bladder cancer cells (K9TCC-Pu-AXC, K9TCC-Pu-AXA, K9TCC-Pu-NK, K9TCC-Pu-IN) measured by flow cytometry. Expression levels of EGFR and HER-2 represent the relative mean fluorescence intensity (rMFI). The results are shown as means ± standard deviation (SD) measured in triplicate from three independent experiments.


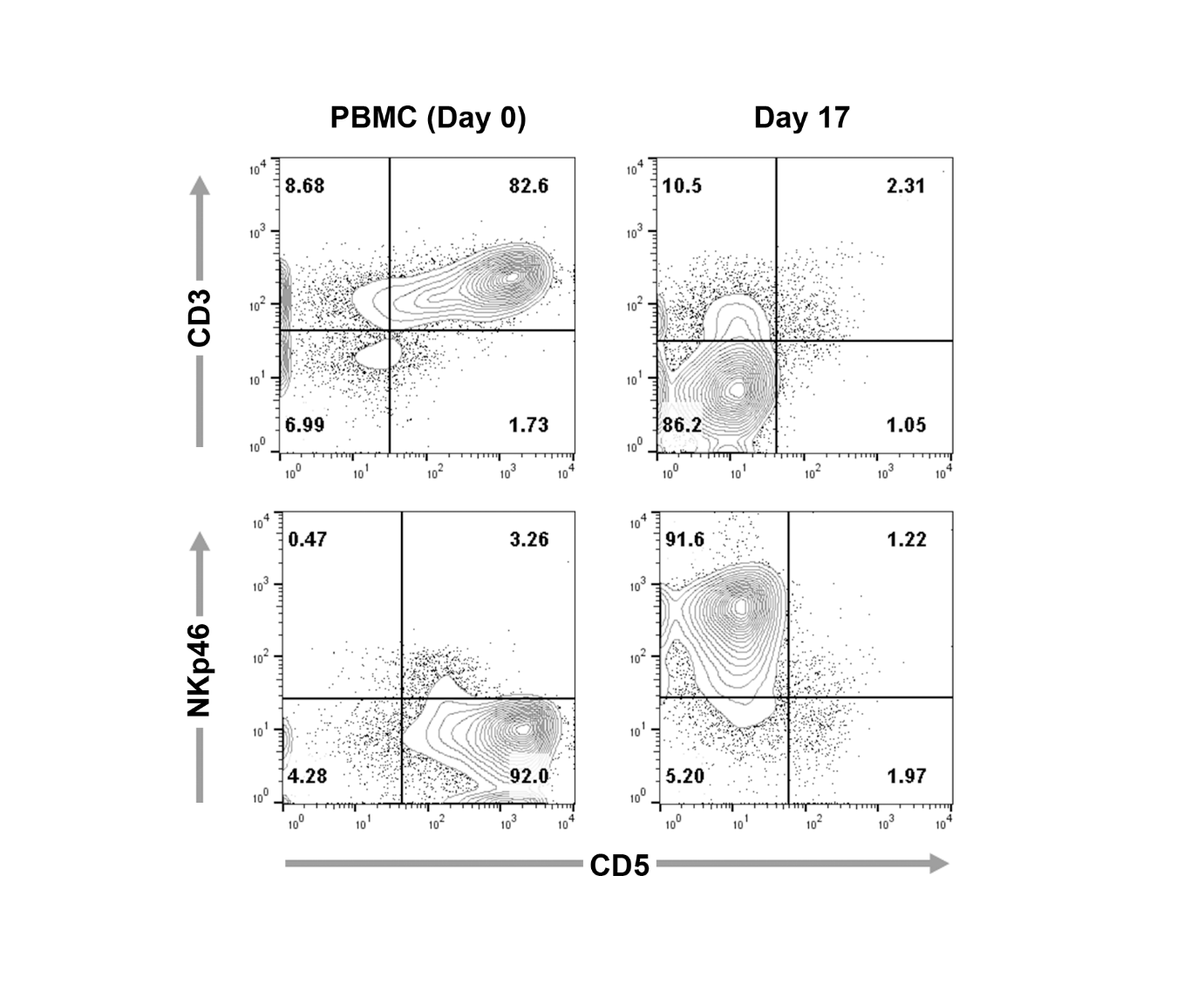


**Supplementary Fig. S2.** Expression of NKp46 on cultured non-B, non-T (CD3^–^ CD5^–^ CD21^–^) NK lymphocytes. PBMCs (Day 0) and expanded NK cells (Day 17) were stained with anti-dog NKp46 antibody (clone 48A). Representative flow cytometry plots showing more than 90% of non-B, non-T NK cells express NKp46 at 17 days after culture (n=4).


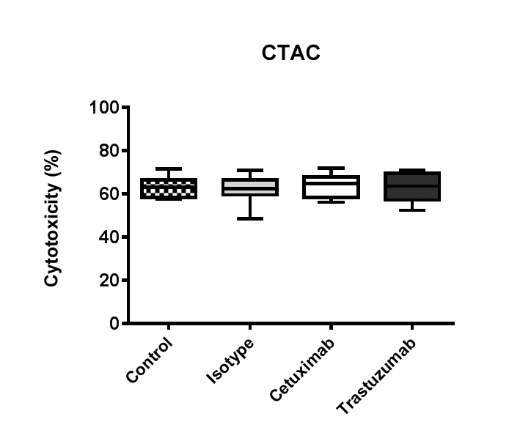


**Supplementary Fig. S3.** The ADCC ability of expanded canine NK cells against trastuzumab- or cetuximab-coated canine thyroid adenocarcinoma (CTAC) cells that do not express HER-2 and EGFR. The 4-h cytotoxicities of NK cells were measured at a 4:1 effector-to-target (E:T) ratio from triplicate reactions and 5 different donors. The cytotoxicity of NK cells against CTAC cells pretreated with media alone or human isotype IgG antibody served as controls. The median, first (Q1) and third (Q3) quartiles, and the minimum and maximum are shown.


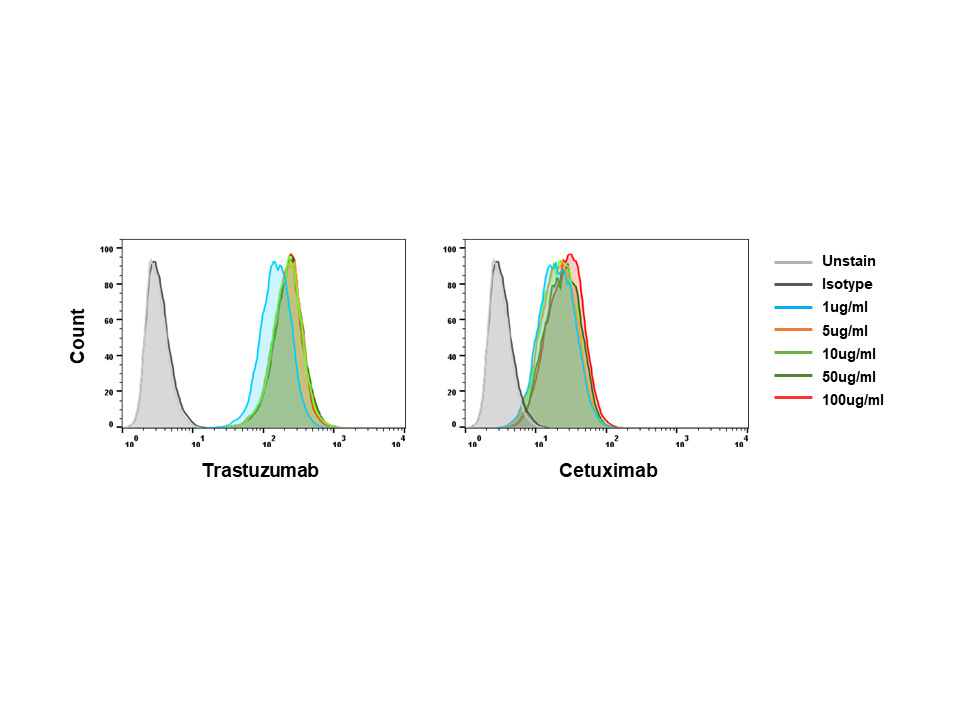


**Supplementary Fig. S4.** Binding of trastuzumab and cetuximab to SKBR3 cells by flow cytometry. Both trastuzumab and cetuximab were tested at 1, 5,10, 50 and 100 μg/ml. Cells unstained and stained with human IgG isotype antibody were used as controls. The results shown represent those from three independent experiments, each performed in triplicate.

**Supplementary Methods**

**Cell lines and monoclonal antibody.** The canine urinary bladder cancer cell lines K9TCC-Pu-AXC, K9TCC-Pu-AXA, K9TCC-Pu-NK, and K9TCC-Pu-IN were kindly provided by Prof. Deborah W. Knapp (Department of Veterinary Clinical Science, Purdue University, West Lafayette, IN, USA). Canine thyroid adenocarcinoma (CTAC) cells (i.e., canine NK cell sensitive cells) were obtained from the European Collection of Cell Culture (Porton Down, UK). The canine mammary gland tumor cell lines CMT-U309 and CMT-U336 were kindly provided by Prof. Eva Hellmen (Swedish University of Agricultural Sciences, Uppsala, Sweden). Anti-canine NKp46 antibody (clone 48A) was kindly provided by Prof. Dean A. Lee (Department of Hematology, Oncology, and Bone Marrow Transplantation, Nationwide Children's Hospital, Columbus, OH, USA).

**Binding assay for trastuzumab and cetuximab**. To determine the optimal concentrations of trastuzumab and cetuximab for ADCC assay, SKBR3 cells (2 × 10^5^) were incubated with 1, 5, 10, 50, and 100 μg/ml of cetuximab (Merck KGaA) or trastuzumab (Roche) for 15 min on ice. The cells were then washed three times with FACS buffer (phosphate-buffered saline, 5% bovine serum albumin) and incubated with 2 μl of Alexa Fluor 488-conjugated goat anti-human IgG antibody (Southern Biotech) for 15 min on ice. After washing twice with FACS buffer, FACS analysis was performed using the FACSCalibur flow cytometer (Becton Dickinson).

1. The present address of Yoseop Kim is Research Institute, Vaxcell-Bio Therapeutics, Hwasun, Jellanamdo, Republic of Korea

   The present address of Soo-Hyeon Lee is CHABiolab Co.,Ltd ,Seongnam-si ,Gyeonggi-do ,Republic of Korea [↑](#footnote-ref-1)
